# Dimensional Ridge Preservation with a Novel Highly Porous TiO_**2**_ Scaffold: An Experimental Study in Minipigs

**DOI:** 10.1155/2012/851264

**Published:** 2012-10-03

**Authors:** Hanna Tiainen, Anders Verket, Håvard J. Haugen, S. Petter Lyngstadaas, Johan Caspar Wohlfahrt

**Affiliations:** Department of Biomaterials, Institute for Clinical Dentistry, University of Oslo, PO Box 1109, Blindern, 0317 Oslo, Norway

## Abstract

Despite being considered noncritical size defects, extraction sockets often require the use of bone grafts or bone graft substitutes in order to facilitate a stable implant site with an aesthetically pleasing mucosal architecture and prosthetic reconstruction. In the present study, the effect of novel TiO_2_ scaffolds on dimensional ridge preservation was evaluated following their placement into surgically modified extraction sockets in the premolar region of minipig mandibles. After six weeks of healing, the scaffolds were wellintegrated in the alveolar bone, and the convex shape of the alveolar crest was preserved. The scaffolds were found to partially preserve the dimensions of the native buccal and lingual bone walls adjacent to the defect site. A tendency towards more pronounced vertical ridge resorption, particularly in the buccal bone wall of the nongrafted alveoli, indicates that the TiO_2_ scaffold may be used for suppressing the loss of bone that normally follows tooth extraction.

## 1. Introduction

Oral rehabilitation with dental implants requires sufficient vertical and horizontal dimensions of the residual alveolar ridge in order to accommodate and fully embed the endosteal implants in alveolar bone [1, 2]. However, the dynamic hard tissue remodelling that occurs following tooth extraction typically results in significant resorption of the residual alveolar ridge, and the rate of this progressive postextraction bone loss has been shown to be fastest during the first three months [3, 4]. Since the loss of bone height and original ridge contour can lead to complications in implant placement, reducing the dimensional ridge alterations plays an important role in achieving mechanically stable and aesthetically pleasing clinical outcome in implant-retained prosthetic rehabilitation.

Several clinical and preclinical studies have described the morphological changes occurring in the alveolar process in both apical-coronal and buccal-lingual directions following extraction of teeth [2, 3, 5, 6]. The healing sequence of an alveolar socket begins with the formation of a blood coagulum within the alveolus, which subsequently formed into a temporary matrix before osteoblastic reorganisation of the temporary matrix to woven bone [7]. Simultaneously, the socket walls undergo bone remodelling and osteoclastic resorption, and it has been shown that particularly the buccal aspect of the residual ridge is susceptible to reduction in bone volume [6]. It has been suggested that immediate implant placement in fresh extraction sockets can help maintain the original ridge shape [8], but remodelling in the later stages of the healing sequence still results in marked reduction in the buccal bone volume as has been shown in experimental studies by Araújo et al. [9, 10]. This finding is in good agreement with recent clinical studies reporting that immediate implant placement fails to prevent the lateral resorption of the alveolar ridge following tooth loss [11, 12]. 

An alternative approach in the attempt of dimensional ridge preservation has been the grafting of the fresh alveoli using porous bone graft substitutes, which ideally both stimulate the healing of the alveolar socket and inhibit the residual ridge resorption [13]. Several experimental and clinical studies have reported promising results in respect of dimensional ridge preservation for various synthetic and animal-derived bone graft substitutes [14–18]. Bioresorbable materials in granulated form have typically been employed as grafting material in extractions sockets where subsequent implant placement is expected [13]. However, because of the low resorption rates of many currently used bone graft substitutes [19, 20], nonresorbable materials may also be considered a reasonable alternative for alveolar ridge preservation, particularly when highly porous materials are used as bone graft substitutes.

Due to its excellent biocompatibility and favourable osteogenic properties, ceramic titanium dioxide (TiO_2_) has been proposed as a promising material for nonresorbable synthetic bone grafts, or scaffolds, in the restoration of large bone defects [21, 22], and highly porous TiO_2_ scaffolds are considered to be suitable for bone augmentation in dental applications. Since the high surface-to-volume ratio and large accessible pore volume provide sufficient space for cell attachment, cell proliferation, and nutrient and metabolite transportation, the highly porous TiO_2_ scaffold structure allows excellent conditions for initial osteoblast attachment and proliferation of human mesenchymal stem cells (hMSC) *in vitro* [23, 24]. Furthermore, the well-interconnected pore structure of the TiO_2_ scaffolds has been shown to allow unobstructed bone ingrowth when placed in fresh, surgically modified extraction sockets in minipig mandibles, which together with the good osteoconductivity of the scaffold material resulted in good mechanical interlocking of the scaffold structure and the newly formed bone tissue within the alveoli [25]. Because of this mechanical stability of the scaffold structure in the alveoli, the porous TiO_2_ scaffolds may also have the potential to inhibit the residual ridge resorption. Hence, the aim of the present animal study was to assess the hard tissue remodelling in the alveolar ridge following tooth extraction in order to establish the effect of the highly porous TiO_2_ scaffold structure on dimensional ridge preservation in minipig mandibles.

## 2. Materials and Methods

### 2.1. Scaffold Production

Ceramic TiO_2_ scaffolds were fabricated by replication process. TiO_2_ slurry was prepared by dispersing 65 g of TiO_2_ powder (Kronos 1171, Kronos Titan GmbH, Leverkusen, Germany) in 25 mL sterilised H_2_O, and the pH of the dispersion was kept at 1.5 for the entire duration of the stirring with small additions of 1 M HCl. The stirring was continued for 2.5 h at 5000 rpm. Cylindrical polyurethane foam templates, 5 mm in diameter and 12.5 mm in height (60 ppi, Bulbren S, Eurofoam GmbH, Wiesbaden, Germany), were coated with the prepared slurry. Prior to sintering at 1500°C for 40 h, the polymer sponge was carefully burnt out of the green body at a lower temperature. After sintering, the scaffolds were recoated with slurry that was prepared using the previously described procedure, only this time mixing 40 g of TiO_2_ powder with 25 mL of water. The recoated scaffolds were then sintered in 1500°C for 24 h. For more details, see [22]. The final dimensions of the scaffolds were 4 mm in diameter and 10 mm in height due to the shrinkage during the sintering phase. The scaffold samples were steam sterilised in 121°C for 15 minutes.

### 2.2. Animals and Surgical Procedures

Six female Göttingen minipigs (Sus scrofa, Ellegaard A/S, Dalmose, Denmark) aged 18 to 24 months and weighing 32–42 kg were acclimatised in the local animal facilities (Malmö General Hospital (MAS), Malmö, Sweden) for two weeks before the surgical procedure. Preparation of animals as well as animal management and care followed routine protocols approved by the Institutional Review Board of MAS. Ethical approval for the experiment had been obtained from the Institutional Review Board at MAS Animal Experimental Ethical Committee Malmö-Lund M130-06.

The animals were maintained under general anaesthesia according to a standard procedure using Ketalar 50 mg/mL (Pfizer, Sollentuna, Sweden) and Midazolam-Hameln 5 mg/mL (Hameln Pharma plus GmbH, Hameln, Germany). After visual inspection of the animals, the pigs were shaved around the mouth, and the skin was then rinsed with chlorhexidine solution (chlorhexidine 5 mg/mL in 60% EtOH, Apoteksbolaget, Stockholm, Sweden). 

**Figure 1:**
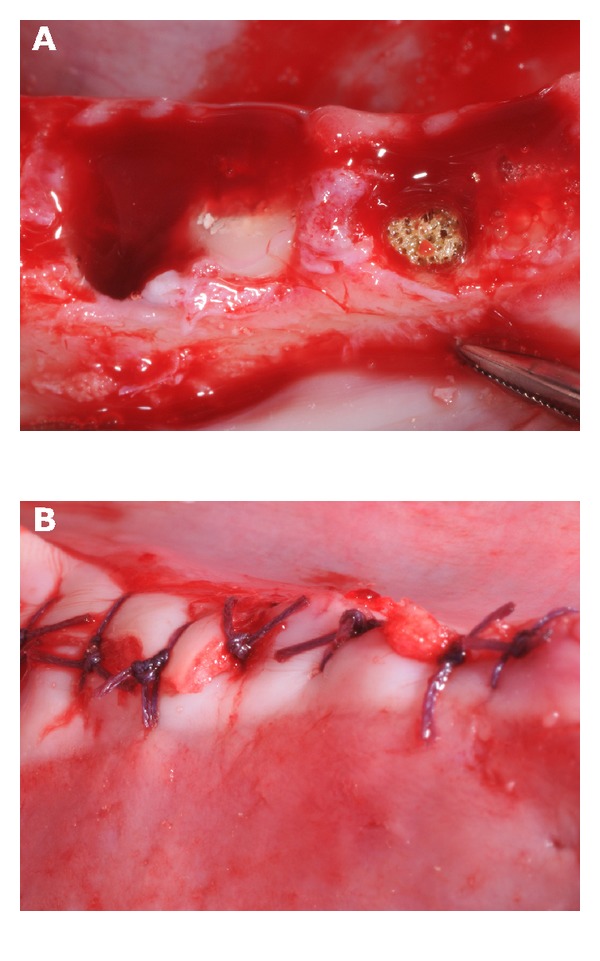
Clinical photographs illustrating the sham and scaffold sites (A) and the socket sites covered by mucosa flaps that were retained in position with interrupted sutures (B).

**Figure 2:**
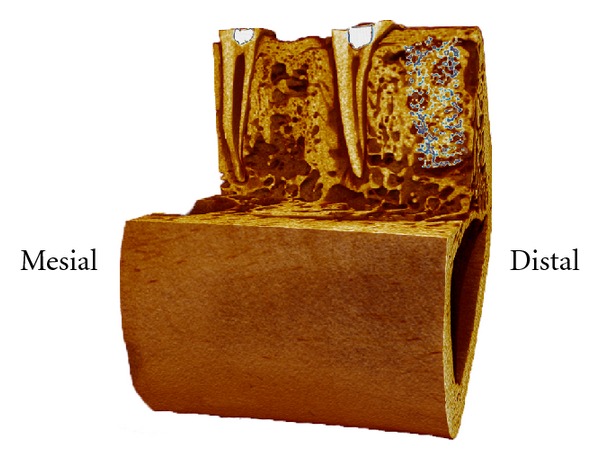
Representative three-dimensional illustration of the scaffold (right) and sham (left) sites with their corresponding mesial roots after six weeks of healing (reconstructed from micro-CT data using CTvox).

Buccal and lingual incisions were made in the posterior premolar region in both quadrants of the mandibles. Buccal and lingual full-thickness flaps were elevated to expose the alveolar crest. Two premolars (P_3_ and P_4_) were hemisected on both sides using fissure burs, and the distal roots were carefully removed using elevators and forceps. The extraction sockets were surgically treated to accommodate the cylindrical TiO_2_ scaffold using burs 4 mm in diameter resulting in standardised cylindrical defects 10 mm deep and 4 mm in diameter. The resulting defects were either grafted with a porous TiO_2_ scaffold or left untreated (sham) in alternate pattern so that each mandible contained equal number of scaffold and sham sites (Figures 1 and 2, *n* = 12). 

The surgical sites were closed with Vicryl 4.0 resorbable sutures (Polyglactin 910, Ethicon Inc., Somerville, NJ, USA). Particular care was taken to ensure that the flap completely covered the extraction sites and that passive stability of the flap had been obtained. The same procedure was repeated for both quadrants of the mandible. 

The animals were kept on soft diet for one week after surgery. Antibiotic (Streptocillin vet 250 mg/mL + 200 mg/mL Boehringer Ingelheim GmbH, Ingelheim, Germany) was administered for seven days after surgery, and 1 mL/10 kg analgesics (Temgesic 0.3 mg/mL Schering-Plough, Brussels, Belgium) was administered once after surgery. Six weeks after surgery, a lethal injection of 40 mL pentobarbital natrium 100 mg/mL in spiritus fortis 290 mg/mL was given intracardially to the animals. 

Segments including all experimental teeth were excised en bloc. After 48 hours of fixation in formaldehyde, the biopsies were transferred to 70% alcohol, and the bone segments were later sequentially embedded in light-curing epoxy (Technovit 7200 VLC, Heraeus Kulzer GmbH, Wehrheim, Germany). All procedures were documented with clinical photographs.

### 2.3. Micro-Computed Tomography (Micro-CT)

Bone formation and dimensional ridge alterations were analysed using micro-CT imaging (SkyScan 1172 high-resolution micro-CT, SkyScan N.V., Kontich, Belgium). All samples were scanned at 17.1 *μ*m voxel resolution using a source voltage of 100 kV and a current of 100 *μ*A with 0.5 mm aluminium filter to optimise contrast. Samples were rotated 360° around their long axes, and four absorption images were recorded every 0.4° of rotation. These raw images were then reconstructed with the standard SkyScan reconstruction software (NRecon) to serial coronal-oriented tomograms using 3D cone beam reconstruction algorithm. For reconstruction, beam hardening was set to 40% and ring artefact reduction to 16. The image analysis of the reconstructed axial bitmap images was performed using standard SkyScan software (CTAn and CTVox).

**Figure 3:**
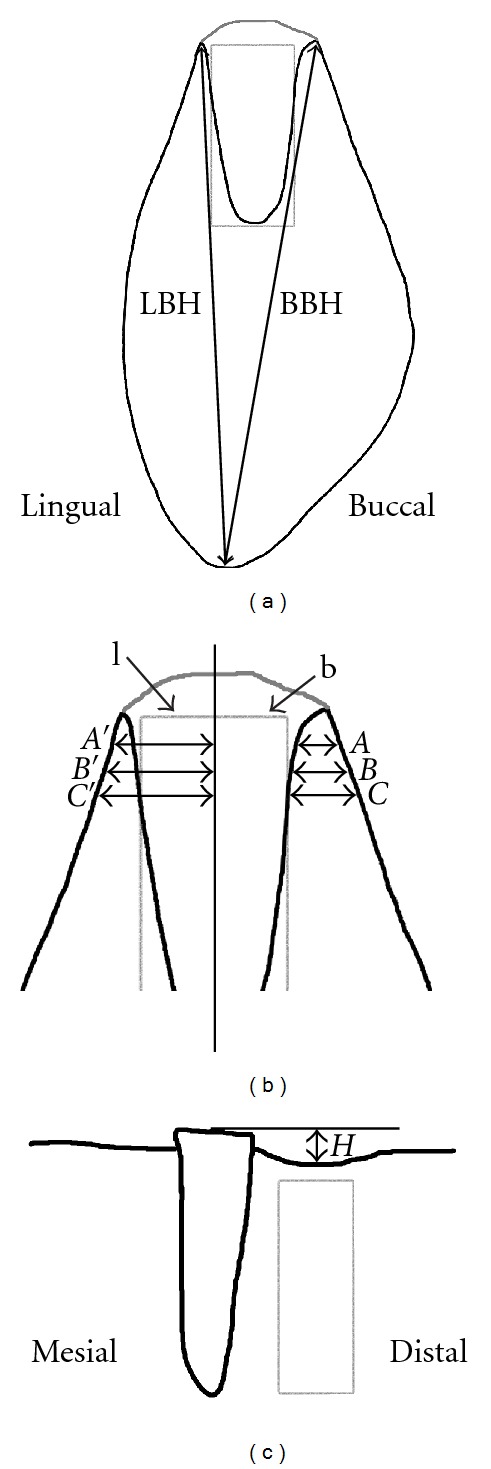
Schematic drawing representing the locations of the morphometric measurements performed in this study with the grey rectangle showing the approximate shape and place of the scaffolds/shams. (a) Vertical parameters: buccal bone height (BHH) and lingual bone height (LBH). For scaffold and sham sites, a straight line was drawn along the edges of the scaffold/sham, and bone height was measured where this line intersected the bone crest. In addition, the vertical height difference between buccal and lingual bone crest was measured (lines b and l in (b)). (b) Horizontal parameters: buccal and lingual alveolar wall width (*A*, *B*, *C*) and buccal and lingual total bone width (*A*′, *B*′, *C*′) 1, 3, and 5 mm apical of the buccal and lingual bone crest, all measured perpendicular to the long axis of the defect. (c) Vertical bone loss: difference in height (*H*) between original bone level and bone level at defect site after six weeks of healing.

Reconstructed cross-sectional images were recorded from the central area of each remaining root as well as their corresponding alveolus in both buccal-lingual and mesial-distal plane. Three buccal-lingual micro-CT sections from each root and alveolus were recorded, and the following vertical and lateral aspects of the root/defect sites were evaluated: buccal and lingual total bone height (BBH, LBH) measured from the top of the buccal and lingual alveolar wall to the most apical point of the jaw (Figure 3(a)), buccal and lingual alveolar wall bone width (*A*, *B*, *C*), and total bone width (*A*′, *B*′, *C*′) measured perpendicular to the long axis of the root/defect 1, 3, and 5 mm underneath the top of the alveolar crest (Figure 3(b)). In addition, the vertical height difference between the most coronal positions of the buccal and lingual bone crest (b-l) was measured (Figure 3(b)). Each measurement for sites containing the remaining mesial root was subtracted from the equivalent measurement on the corresponding distal extraction site. The shape and dimensions of the alveolar process at the mesial and distal roots were presumed to be similar. From the mesial-distal sections, the vertical bone loss at the alveolar bone crest was measured in comparison with the original bone level marked by the top of the remaining mesial root (Figure 3(c)). 

One mandible segment containing one scaffold and one sham site was excluded from the micro-CT analysis due to damage during sample preparation.

### 2.4. Histological Examination

Histological sections were prepared according to the cutting-grinding technique described by Donath and Breuner and Rohrer and Schubert [26, 27]. One central buccal-lingual section of each scaffold and sham site was prepared. Approximately 100 *μ*m thick sections were stained with haematoxylin and eosin for light microscopy and digital imaging. The bone tissue adjacent to the defect area (scaffold and sham) was examined using Leitz DMRBE microscope (Leica, Wetzlar, Germany) equipped with Cell^B^ imaging system (Olympus Soft Imaging Solutions GmbH, Münster, Germany). All sections were evaluated qualitatively for the appearance of the alveolar ridge adjacent to the scaffold and sham sites as well as inflammation and/or immunological reactions in the extraction sockets. 

### 2.5. Statistical Analysis

The normality and equal variance of the datasets were tested prior to further statistical analysis. When datasets were found normally distributed, statistical analyses between the datasets were performed using Student's *t*-test, while the datasets that failed the normality or equal variance test were analysed using nonparametric Mann-Whitney *U* test. Statistical significance was considered at a probability *P* < 0.05. *Post hoc *power calculations were performed for all analyses. All statistical analyses were performed using SigmaStat software package (SigmaStat v. 3.5, Systat Inc., St. Louis, USA).

## 3. Results

### 3.1. Clinical Observations

All defect sites demonstrated uneventful healing without clinical signs of inflammation at the time of harvest. 

### 3.2. Histological Examination

**Figure 4:**
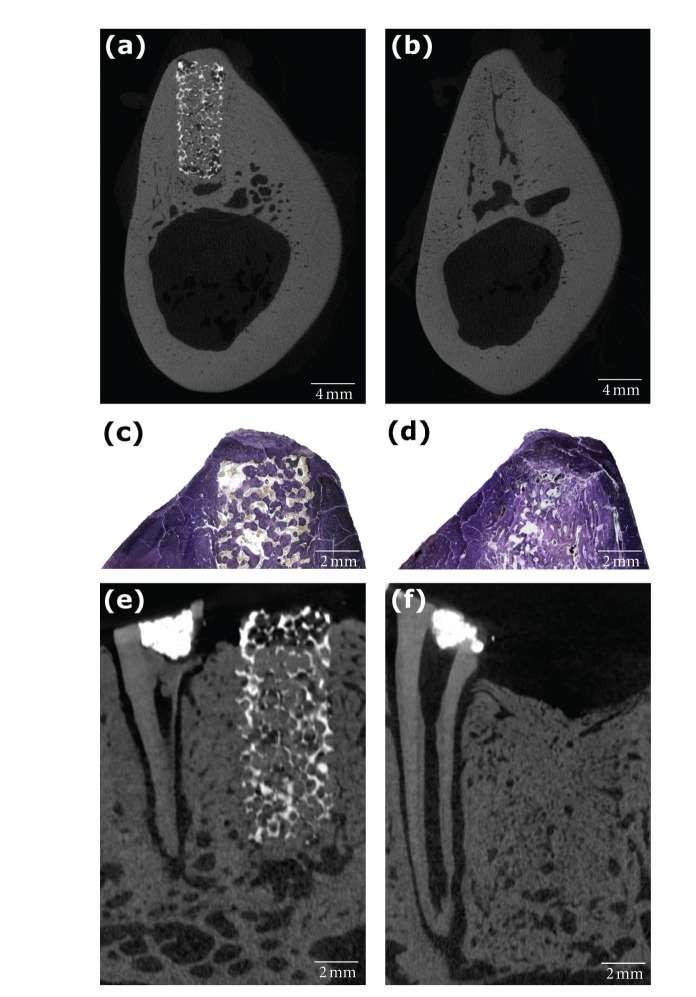
The scaffolds were well integrated in the alveolar bone, and the TiO_2_ scaffold was not found to interfere with the normal healing sequence of the extraction socket (a–d). In few isolated instances (2/11), small portion of the scaffold was exposed above the newly formed bone (e), while the loss of ridge height at a sham site is shown in (f).

After six weeks of healing, the morphological appearance of the scaffold, and sham sites did not differ markedly. Both defect sites were sealed by newly formed hard tissue consisting of dense woven bone giving a dome-shaped outline for the bone crest of the defect sites in the buccal-lingual sections. Typically, no distinct border that separated the newly formed bone from the old cortical bone of the buccal and lingual walls could be identified in the histological sections. For the majority of the scaffold sites, a bony bridge with the average height of 1.23 ± 0.35 mm had formed above the scaffold material (Figures 2 and 4) as measured from the central mesial-distal cross-sections. In few isolated instances (2/11) which both were in the same individual, there was no bony bridge covering the entire scaffold and a small portion of the scaffold was exposed above the newly formed bone (Figure 4(e)). The porous scaffold structures were well integrated in the newly formed bone tissue in the alveolar bone, and the TiO_2_ scaffolds were not found to interfere with the normal healing sequence of the extraction socket (Figures 2 and 4). Both scaffold and sham sites showed a large bone volume within the alveoli, and only a small part of the alveolar ridge was occupied by loose connective tissue.

### 3.3. Dimensional Ridge Alterations

**Figure 5:**
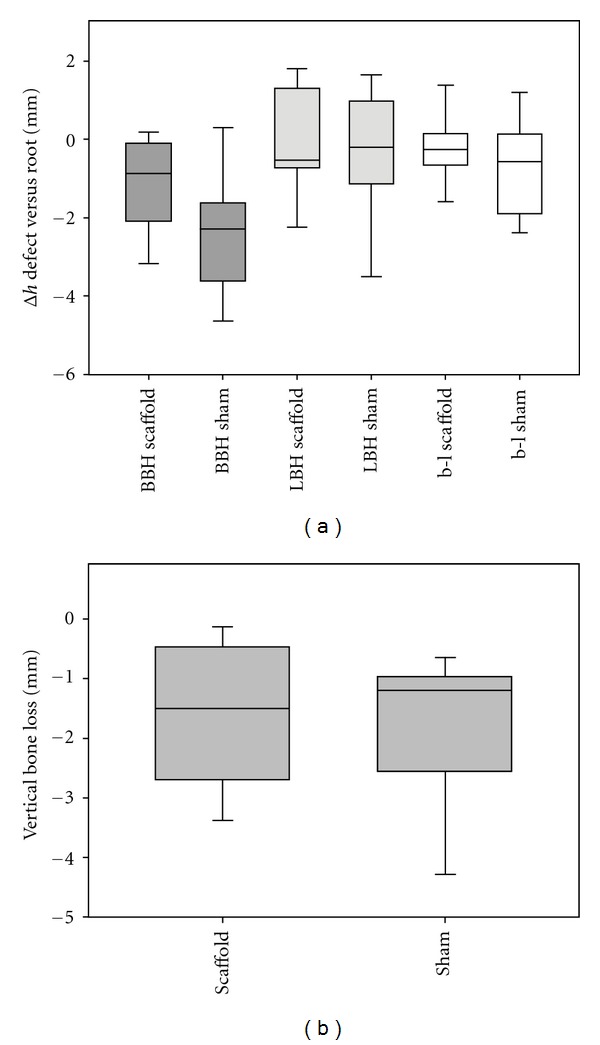
(a) Box plot showing the medians and distributions of the difference in buccal and lingual bone height (BBH and LBH) and height difference of buccal and lingual bone crest (b-l) in comparison to equivalent measurement on the corresponding mesial root of each defect site (Δ*h* = defect − root). (b) Box plot of vertical bone loss relative to the original bone level measured from central micro-CT sections cut in the mesial-distal direction. The whiskers of the plots represent the 5th and 95th percentiles, *n* = 11.

**Figure 6:**
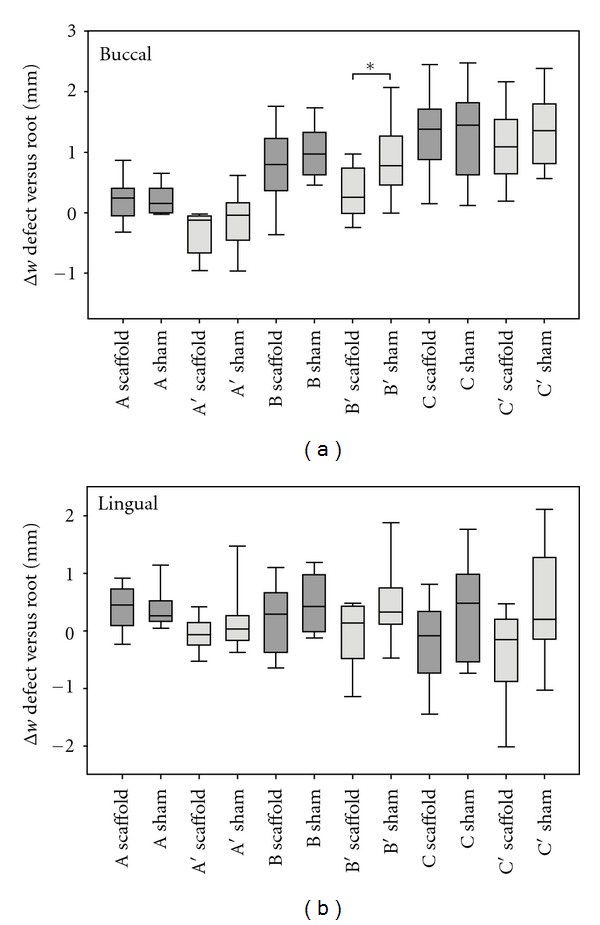
Box plot showing the medians and distributions of difference in buccal and lingual alveolar wall (*A*–*C*) and total bone thickness (*A*′, *C*′) in comparison to equivalent measurement on the corresponding mesial root of each defect site 1 mm (*A*), 3 mm (*B*), and 5 mm (*C*) apical of the buccal and lingual bone crest (Δ*w* = defect − root). The whiskers of the plot represent the 5th and 95th percentiles, *statistically significant difference in this parameter (*P* < 0.05), *n* = 11.

The medians and interquartile ranges of the difference in buccal and lingual bone height (BBH and LBH) and height difference of buccal and lingual bone crest (b-l) in comparison to equivalent measurement on the corresponding mesial root of each defect site (Δ*h* = defect − root) as well as the vertical bone loss are shown in Figure 5. The difference in BBH was noticeably smaller for the scaffold group although no statistically significant difference was found between the scaffolds and shams. The results of the horizontal morphological measurements are presented in Figure 6 as medians and interquartile ranges of the difference in each parameter in comparison to the equivalent measurement on the corresponding mesial root of each defect site (Δ*w* = defect − root). No morphometric aspect apart from *B*′ (buccal) revealed statistically significant difference between the scaffold and sham sites (*P* > 0.05). However, the power of the performed comparisons between each parameter was below 80%. Both vertical and horizontal parameters were also compared without subtracting the equivalent measurement of the corresponding mesial root (Tables 1 and 2). Apart from the buccal bone height, no statistically significant difference in any of the measured parameters was detected (*P* > 0.05) although the power of the performed comparisons was below the desired power of 80% for all aspects. 

## 4. Discussion

This is the first study to investigate the effect of the highly porous TiO_2_ scaffold construct on the dimensional ridge alterations occurring following tooth extraction. The highly porous TiO_2_ scaffolds were well integrated in the alveolar bone with a dense bony bridge covering the entire scaffold at the most coronal point of the ridge (Figures 2 and 4) six weeks following their implantation in extraction sockets in the premolar region of minipig mandibles. The excellent osteoconductive capacity of TiO_2_ scaffolds has previously been reported by the present authors [25]. It was shown that the scaffold structure permits excellent bone tissue penetration to the entire pore volume when placed in fresh, surgically modified extraction sockets, and no significant delay in the healing of the socket was observed in comparison to empty sockets during the six-week healing period. The bone mineral density was also found higher for sockets containing TiO_2_ scaffolds in comparison to the empty control sockets, thus indicating improved bone matrix mineralisation in the presence of the TiO_2_ scaffold in mandibular extraction sockets [25]. Furthermore, it is likely that the porous TiO_2_ scaffold can advance the closure of the marginal entrance of the alveolus with newly formed bone tissue, as indicated by the large volume of bone tissue observed above the scaffold structure for great majority of the grafted alveoli (Figure 4(a)). Early closure of the socket is desired in order to seal the defect site from soft tissue proliferation and invagination into the extraction socket as the ingrowth of endothelial tissue may result in reduced bone fill within the alveolus [13]. For this purpose, bone grafts and bone graft substitutes are sometimes used in combination with a guided tissue regeneration (GTR) membrane in order to isolate the alveolus from the soft tissue, and thereby improve the maintenance of the ridge dimension [28]. Since the scaffold was completely embedded in newly formed bone tissue and enclosed by the marginal bone at great majority of the scaffold sites, there was no surface invagination of marginal bone, and the convex shape of the alveolar crest was preserved also in the absence of GTR membrane. However, it appears that the TiO_2_ scaffold does not have the capacity to fully prevent the loss of bone height at the defect site in the present animal model. When the vertical bone loss was measured from the central mesial-distal sections, no marked difference in the lost bone height was observed between the grafted and nongrafted sites (Figure 5(b)). This may be related to the cylindrical dimensions of the experimental TiO_2_ scaffolds. While particulate and pastelike bone grafts can easily be made to fill the entire contour of the alveolus, with preformed rigid scaffold constructs; this can only be achieved if the alveolus can be shaped to fit the scaffold dimensions or vice versa. Since the scaffold structure had a predefined shape and dimensions that did not mimic the contours of the extraction alveoli, the TiO_2_ scaffold did not always fill the entire defect volume close to the marginal entrance of the socket. If the graft material does not reach the original bone level or leaves a gap between the scaffold and alveolar walls, it cannot provide sufficient mechanical support to the adjacent bone walls at the most marginal regions of the alveoli, which is likely to result in loss of bone height at the alveolar ridge as seen in the present study. 

Various studies have suggested that bone graft substitutes can suppress the residual ridge resorption, while others have questioned the efficacy of bone graft substitutes in preserving the original contour of the alveolar ridge. Several experimental studies in dogs have reported a favourable outcome in regard to dimensional ridge preservation when fresh extraction sockets were grafted with various bone graft substitute materials [17, 29–31]. The effect is commonly attributed to the capacity of the grafting material to provide a scaffold for new bone formation and to offer stability for the blood coagulum that forms in the initial stages of the healing process [15]. It appears that good osteoconductive capacity alone cannot serve to preserve the dimensions of the edentulous alveolar ridge, as grafting the sockets with highly osteogenic but fast resorbing autologous bone chips was not shown to prevent the ridge resorption following tooth extraction [32]. Moreover, other studies with similar experimental setups have concluded that various bone graft substitute materials fail to preserve the alveolar ridge height [33–35]. Fickl et al. reported that incorporation of deproteinised bovine bone xenografts (DBBX) into fresh extraction sockets has only a limited impact on the resorption of the buccal plate [34]. Similarly, a recent study by Bashara et al. showed no statistically significant difference in the residual ridge resorption of empty extraction sockets and sockets grafted with either DBBX or porous titanium granules both with and without an additional GTR membrane after 6-month healing period [33]. 

While the excellent osteoconductive capacity of the pore network of the TiO_2_ scaffolds can provide a favourable environment for bone regeneration within the extraction socket, the mechanical stability of the TiO_2_ scaffold block is expected to give good initial structural stability to the adjacent socket walls during the early phases of the healing sequence. However, it appears that the scaffold did not fill the entire defect volume as already mentioned above, and there was no conclusive evidence for the TiO_2_ scaffold to prevent the reduction in the alveolar ridge dimensions. A statistical significance was observed in the buccal bone heights between scaffold and sham sites, while a more prominent reduction in buccal bone height in comparison to corresponding distal alveoli was also evident for the nongrafted extraction sockets as illustrated in Figure 5(a). However, no significant difference in the lateral resorption was apparent between the grafted and no-grafted sockets apart from the statistical significance in the buccal total bone width 3 mm apical from the alveolar crest (Figure 6). This difference was not found statistically significant when the actual horizontal bone widths at this level were compared, but all horizontal aspects at 3 and 5 mm apical from both buccal and lingual crest were found noticeably larger for the nongrafted group (Table 2). This was also observed in Figure 6 depicting the difference in bone width, particularly for the lingual bone wall. As the horizontal measurements were performed in respect to the buccal and lingual bone crests, the measured lateral dimensions are strongly affected by the vertical resorption with increasing vertical bone loss resulting in larger measured bone width values. Therefore, the larger apparent increase in the alveolar bone width in the sham group can be seen as an indication of the resorption of alveolar crest and loss of ridge height. Still, the lack of marked difference in the lingual height difference for scaffold and sham sites (Figure 5(a)) seems to be in disagreement with this notion. Nevertheless, the observed tendency towards more pronounced vertical ridge resorption in the buccal bone wall of the nongrafted alveoli may indicate that the TiO_2_ scaffold can suppress the postextraction resorption of the buccal bone wall. In addition, it must be noted that the dimensional ridge alterations were recorded after a relatively short healing period of six weeks, even if the bone resorption has been shown to occur at a fast rate during the first three months [3]. Longer observation periods may result in more discernible difference in the ridge of the grafted and nongrafted sites, and therefore further experimental studies with multiple healing periods are recommended to fully characterise the effect of the TiO_2_ scaffolds in preserving the alveolar ridge dimensions. 

Inaccuracies and technical complications in the evaluation of the dimensional ridge alterations may also have influenced the outcome of the comparisons between grafted and nongrafted sites. In the present study, all dimensional aspects of the alveolar ridge were measured from the cross-sectional micro-CT images rather than the histological sections as the nondestructive digital sectioning allowed more precise alignment of the cross-sectional images to the centre of each defect than what could be achieved by the cutting and grinding method used for the production of histological sections. For scaffold sites, the radiopaque TiO_2_ structure clearly defined the lateral boundaries of the socket, and thus, the lateral endpoints of the measurements were easily distinguished. The same applies for root sites in which the remaining roots distinctly defined the boundaries of the alveolar socket. However, this was not the case for the sham sites as the boundaries of the original alveolus were not always clearly visible in either micro-CT cross-sections or histological slide as can be seen in Figure 4. Therefore, defining the end-points for the horizontal measurements was not always straightforward for the nongrafted extraction sockets, and may have resulted in slightly erroneous width measurements. In addition, the measurements applied here are further restricted by the two-dimensional nature of the measurements which may partially omit the changes in the true three-dimensional ridge contour.

Moreover, the comparison of the different experimental animal studies performed to evaluate the effect of a given bone graft material in preserving the original ridge dimensions following tooth extraction is somewhat restricted due to the variation in the measured parameters and measurement techniques used in each study. Using the distal alveolus with the remaining root as a reference for each experimental site, as described by Araújo et al. [29], allows fairly good evaluation of the amount of ridge contraction in comparison to the original ridge dimensions. However, this method relies on the presumption that the mesial and distal alveoli, as well as the adjacent bone walls, have the same shape. In addition, it must be assumed that only minimal resorption occurs at alveolar walls adjacent to the root sites. To adjust to these assumptions, the results of dimensional measurements of the sham and scaffold sites were also compared to each other without the subtraction of the ridge dimensions measured for the mesial root sites. These results were mostly in line with the results obtained by calculating the dimensional differences, thus reinforcing the conclusions of the present study. However, it is important to keep these limitations in mind when interpreting the results of this study. 

## 5. Conclusions

The TiO_2_ scaffolds were completely embedded in newly formed bone tissue, and the convex shape of the alveolar crest was preserved. However, it appears that the TiO_2_ scaffold does not have the capacity to fully prevent the loss of alveolar bone height following tooth extraction in the present animal model. Nevertheless, the observed tendency towards more pronounced vertical ridge resorption particularly in the buccal bone wall of the nongrafted alveoli indicates that the TiO_2_ scaffold may suppress the postextraction resorption of the buccal bone wall. Due to the short healing period of the present study, further experimental studies with longer healing periods are recommended to fully characterise the effect of the TiO_2_ scaffolds in preserving the alveolar ridge dimensions. 

## Figures and Tables

**Table 1:** Mean values and standard deviations of buccal and lingual bone height (BBH and LBH, respectively) in millimetres after 6 weeks of healing.

	Scaffold	Sham	*P* value
BBH	35.36 ± 1.63	33.51 ± 1.72	0.02*
LBH	34.52 ± 1.68	33.80 ± 2.37	0.45
BBH-LBH	0.84 ± 1.13	−0.29 ± 1.43	0.07

*Significant difference between the two groups *P* < 0.05. *n* = 11.

**Table 2:** Mean values and standard deviations of alveolar wall thickness (*A*–*C*) and total bone thickness (*A*′ –*C*′) on different levels: 1 mm (*A*), 3 mm (*B*), and 5 mm (*C*) apical of the buccal and lingual bone crest. Values are given in millimetres after 6 weeks of healing. No significant difference was detected between scaffold and sham groups. *n* = 11.

	Scaffold	Sham	*P* value		Scaffold	Sham	*P* value
*A* buccal	0.85 ± 0.43	0.81 ± 0.29	0.82	*A*′ buccal	2.98 ± 0.52	3.22 ± 0.58	0.35
*B* buccal	2.01 ± 0.84	2.31 ± 0.66	0.37	*B*′ buccal	4.11 ± 0.93	4.67 ± 0.94	0.20
*C* buccal	3.28 ± 1.14	3.80 ± 0.93	0.28	*C*′ buccal	5.35 ± 1.22	6.17 ± 1.12	0.14

*A* lingual	0.84 ± 0.43	0.77 ± 0.35	0.69	*A*′ lingual	2.93 ± 0.53	3.15 ± 0.58	0.41
*B* lingual	1.64 ± 0.56	1.73 ± 0.52	0.73	*B*′ lingual	3.74 ± 0.68	4.09 ± 0.69	0.27
*C* lingual	2.37 ± 0.61	2.54 ± 0.63	0.53	*C*′ lingual	4.43 ± 0.69	4.89 ± 0.79	0.19
